# Providing culturally safe cancer survivorship care with Indigenous communities: study protocol for an integrated knowledge translation study

**DOI:** 10.1186/s40814-019-0422-9

**Published:** 2019-02-26

**Authors:** Wendy Gifford, Roanne Thomas, Gwen Barton, Ian D. Graham

**Affiliations:** 1 School of Nursing, Faculty of Health Sciences, University of Ottawa, 451 Smyth Road, Ottawa, ON K1H 8M5 Canada; 2 School of Rehabilitation Sciences, Faculty of Health Sciences, University of Ottawa, 451 Smyth Road, Ottawa, ON K1H 8M5 Canada; 3 Indigenous Cancer Program, The Ottawa Hospital Cancer Program, 501 Smyth Rd, Ottawa, ON K1H 8L6 Canada; 4 School of Epidemiology and Public Health, University of Ottawa, 401 Smyth Rd, Ottawa, ON K1H 5B2 Canada; 5 Centre for Implementation Research, The Ottawa Hospital Research Institute, 401 Smyth Rd, Ottawa, ON K1H 5B2 Canada

**Keywords:** First Nations, Inuit, and Métis health, Indigenous people, Cancer survivorship, Implementation science, Integrated knowledge translation, Mixed methods, Community-based participatory research

## Abstract

**Background:**

Cancer among Indigenous people is increasing faster than overall Canadian rates. Lack of survivorship support, including screening and follow-up for recurrences, contributes to poor health outcomes and low 5-year survival rates. Historical trauma from colonization and lack of culturally safe and responsive healthcare has negatively affected Indigenous peoples’ access to survivorship supports. Nurses are typically the sole practitioners of health services in rural and remote Indigenous communities and can enhance the development, implementation, and delivery of culturally safe survivorship supports. However, the implementation of culturally safe healthcare in Indigenous communities is not well developed.

This is the third study in a larger program of research with an overarching goal to improve healthcare delivery and outcomes with Indigenous people in Canada. In this study, we will field test nurses’ implementation of cancer survivorship care with Indigenous people in Ontario, Canada.

**Methods:**

The study is a descriptive participatory mixed methods research design involving a systematic review, field testing implementation of cancer survivorship supports in two communities, focus groups, and qualitative interviews. Outcomes include feasibility of implementation, acceptability of the strategies, and perceived impact on healing and psychosocial support.

**Discussion:**

Results will advance knowledge about implementing culturally safe cancer survivorship supports with Indigenous people in Ontario. A toolkit will be developed to inform nursing practices, programs, and policies to improve cancer survivorship supports and strategies with Indigenous people. Findings will inform a large-scale implementation study to reduce healthcare disadvantages and disparities within Indigenous communities across Canada.

## Background

Cancer mortality is higher among Indigenous people in Canada relative to the general population [1–4]. Although cancer databases in Canada do not have identifiers for race or ethnicity [5], evidence is accumulating that cancer incidents rates for Indigenous people are increasing faster and cancer survival is worse [6, 7]. For example, a recent report to understand cancer prevalence in First Nations people in Ontario has shown that colorectal cancer increased 6 and 7 % respectively for male and female First Nations people, whereas the incidence stayed the same or decreased for non-Indigenous people [5]. Another report has shown that breast cancer among the general population had stabilized but continued to rise for First Nations people [7]. High comorbidities rates and lack of survivorship support such as follow-up screening and secondary prevention contribute to poor health outcomes and lower 5-year survival rates [4–6, 8]. The determinants of cancer mortality and the unique cancer burden faced by Indigenous people in Canada have largely been attributed to the social determinants of Indigenous health such as poverty, social exclusion, racism, discrimination, and lack of culturally responsive healthcare services and supports [2, 5, 9].

Consistent with international health research environments, the term “Indigenous” is used throughout this paper to denote the original inhabitant of a country regardless of its borders. In the Canadian context, Indigenous specifically refers to First Nations, Inuit, and Métis [10]. Almost 1.5 million Indigenous people were living in Canada in 2011, most densely concentrated in the province of Ontario (*n* = 301,425 or 21.5%) [11]. Approximately half this population live on-reserve [11], and 95 out of the 133 Indigenous communities in Ontario are located in rural or remote areas [12]. Nurses are typically the sole practitioners of healthcare services in rural and remote Indigenous communities; as “outsiders,” they often have a poor understanding of the distinct cultural practices, traditions, social and historical roots of health inequities in these settings [13–16].

Although efforts have been made to promote culturally competent care in general nursing educational curricula, nursing knowledge and practice remains largely informed by western culture. Healthcare practices based on western ethnocentric systems have led Indigenous people in Canada to avoid many healthcare services [17]. Lack of awareness and knowledge of culturally safe practices affects nurses’ abilities to implement cancer survivorship strategies and supports that effectively address Indigenous people’s distinct needs.

Through advancements in cancer survivorship care, holistic approaches with long-term follow-up, regular monitoring for early detection, and interventions tailored to specific needs have been shown to increase survival rates and enhance the quality of life for people with cancer [2]. However, interventions predominantly target white, urban, middle-class people, with little done to address the cancer survivorship needs of Indigenous people. Indigenous peoples continue to endure poorer health from a long legacy of marginalization within North America. Metaphorically referred to as “falling through the cracks” [18], little has been done to address the unique survivorship needs of Indigenous people and few supports have considered the intersections of culture, history, and marginalization.

Our research shows that many First Nations people do not seek survivorship support due to the fear of being sent away or of cancer attracting illness and grief into their communities. Moreover, a systematic review of 17 studies revealed that many Indigenous people are prevented from seeking cancer survivorship support by fatalistic attitudes, associated fears, and community stigmatization [19].

This study builds on our foundational studies which overwhelmingly identified a gap in the delivery of culturally safe cancer survivorship support for First Nations and Métis people [18, 20–24]. One of the first opportunities to voice their survivorship experiences, our first study (funded by Canadian Cancer Society), highlighted the profound failures of healthcare services to recognize the distinct ethnic, cultural, and socio-historical positioning of First Nations and Métis people. It revealed that stigmatization, fear of disclosing cancer, and culturally insensitive healthcare approaches prevent Indigenous people from receiving survivorship care, including follow-up and regular monitoring for early detection and survivorship interventions for enhancing quality of life [21, 22, 25, 26].

In our second study (funded by Canadian Institutes of Health Research #356773), we are co-developing cancer survivorship strategies in partnership with First Nation’s healthcare leaders, elders, cancer survivors, and community members, and evaluating the process of developing the strategies to support culturally safe cancer survivorship. Recognizing that each Indigenous community has unique needs, strengths, and cultural traditions, it is unclear how survivorship strategies and implementation approaches in one community will translate to other communities in Canada.

The proposed study is the third in our program of research that has the overarching goal to improve the delivery of culturally safe cancer survivorship care with Indigenous people in Canada. The research questions for this study are the following:

1. What are the cancer survivorship strategies described in the research literature that have been utilized by Indigenous people, and what are the characteristics of collaborative partnerships with Indigenous communities for implementing the strategies identified?
2. What are the barriers and facilitators to nurses implementing cancer survivorship strategies that have been chosen and adapted for cultural safety with Indigenous community members?
3. What is the feasibility and acceptability of implementing cancer survivorship strategies that have been adapted for cultural safety with Indigenous people, and the perceived influence on healing, cancer screening, and psychosocial support?

## Methods

### Design

In collaboration with community partners, a participatory descriptive research design is proposed using multiple methods that include review of literature, focus groups, interviews, and process log.

### Setting and collaboration

The setting involves two communities in the province of Ontario where approximately 41,000 Indigenous people live. One is a rural First Nations community, and one is an urban Inuit community that includes Inuit who have traveled from rural and remote regions of Nunavut for cancer care. Our collaborative partners include nurses, health providers, and program directors from a large urban hospital that strive to implement Cancer Care Ontario’s Aboriginal Cancer Strategy III [12] to deliver culturally relevant and safe cancer care for First Nations, Inuit, and Métis peoples.

Acknowledging that research involving Indigenous people has historically been carried out by non-Indigenous researchers and generally not reflective of Indigenous worldviews (33), we are committed to following ethical principles that recognize strong trusting relationship are integral to ethical research with Indigenous people. Our research is grounded in approaches outlined in Chapter 9 of the Tri-Council Policy Statement (33) that recognizes the importance of traditional cultural values, community engagement, and mutually respectful relationships for conducting research.

Collaboration for the study will be guided by the theoretical underpinnings of integrated knowledge translation (KT) and Indigenous knowledge translation (KT) [10]. The fundamental concept of integrated KT is that researchers and knowledge users, who are patients, practitioners, policy-makers, or members of the public, work together to design and conduct mutually beneficial research [27]. The knowledge that is co-produced through integrated KT may be more meaningful and address real-world knowledge gaps as knowledge users and communities are engaged throughout the entire research process. Indigenous KT recognizes that Indigenous people have a long and established history of creating and translating their own knowledge into actions through oral traditions, engagement, and cross-cultural sharing [28]. Culturally relevant channels of dissemination will be utilized such as kinship networks, talking circles and stories, and participation of community leaders, chiefs, elders, and community members. The continued engagement of community members in our previous study has allowed us to establish partnerships with community leaders, elders, healthcare providers, cancer survivors, and families to plan, design, conduct, and disseminate this study using a participatory, integrated Indigenous KT approach.

### Procedures

#### Research question 1: What are the cancer survivorship strategies described in the research literature that have been utilized by Indigenous people, and what are the characteristics of collaborative implementation partnerships for implementing the strategies identified?

A mixed methods systematic review will integrate evidence from multiple research designs including experimental, quasi-experimental, and observational studies. We will conduct a narrative synthesis to generate insights for policy and practice, and develop hypotheses for future studies [29] as the majority of published research with Indigenous populations is descriptive. A four-stage approach [30] will be utilized to conduct the review.

The search strategy will be shaped by the review question and developed with a health sciences librarian with expertise in systematic reviews. A mix of controlled terminology (MeSH headings) and keywords will be used to capture concepts of engagement, implementation, nurses, healthcare providers, and Indigenous people. No limits will be applied to publication date. Databases will include MEDLINE, CINAHL, Cochrane, Proquest Nursing and Allied Health, ProQuest Dissertations and Theses, ABI Inform Global, and PsycINFO. Studies included in the review will have nurses and other healthcare providers (allied and/or physicians) and a study outcome of cancer survivorship strategies that have been implemented by or in partnership with Indigenous people. Data will be extracted by one reviewer and checked for accuracy by a second; discrepancies resolved through third-party adjudication. Methodological quality of studies will be assessed using specific study design tools (e.g., McMaster Critical Review Form for qualitative research). Data will be aggregated and analyzed narratively for patterns related to cancer survivorship strategies and the implementation process of the strategies. Although not anticipated, if sufficient homogeneity across experimental studies exists, meta-analysis will be performed. Results will be synthesized with survivorship strategies and implementation processes identified in our second study that include the following: healing circles; individual case management with traditional healers, traditional teachers, and elders; creative practices (e.g., beading, sewing, quilting, drawing, journaling); and traditional ceremonies (e.g., prayer, smudging).

A framework will be developed on (1) cancer survivorship strategies for Indigenous peoples; (2) context of implementation, direction, and magnitude of success (if available); (3) characteristics of collaborative partnerships for implementation. Together with the Indigenous community partners, cancer survivorship strategies will be chosen, refined, and adapted for implementation.

#### Research question 2: What are the barriers and facilitators to implementing cancer survivorship strategies that have been chosen and adapted for cultural safety with Indigenous communities?

Qualitative interviews will be conducted with a convenient sample of nurses, nursing aids, and other healthcare providers such as traditional healers working with Indigenous cancer survivors and families. Sample size will be based on data saturation, with 8–10 participants per community to obtain sufficient data. Interviews will be conducted in person (when possible) or by phone, audiotaped, transcribed, and entered into qualitative software (NVIVO-12).

Inductive content analysis will be completed involving data reduction, data display, condensing response categories, and verifying descriptive themes of the barriers and facilitators to implementing cancer survivorship strategies. Consistent with the theories of knowledge translation [31], implementation strategies will be tailored to the identified barriers and facilitators for the greatest chance of success.

#### Research question 3: What is the feasibility and acceptability of implementing cancer survivorship strategies that have been adapted for cultural safety with Indigenous people, and the perceived influence on healing, cancer screening, and psychosocial support?

We will field test nurses’ implementation of the cancer survivorship strategies over 6 months in the two communities.

Sample: based on our previous work, we plan to recruit a purposeful sample of five health providers from each community to implement the cancer survivorship strategies, including nurses, traditional healers, and elders (total *n* = 10). To be included, healthcare providers will have experience working with Indigenous cancer survivors in the community. Together with the research team, the healthcare providers will determine the methods to implement the chosen cancer survivorship strategies, tailoring their implementation methods to the barriers and supports identified (see “Research question 2” section) and the context of the community.

We anticipate 15–20 cancer survivors and caregivers from each community to participate in field testing the intervention for a total of 30–40 cancer survivors and caregivers.

Monthly community visits (*n* = 6) will occur and field notes kept of implementation context. The healthcare providers will keep a process log to longitudinally track the types of survivorship strategies being implemented, the implementation processes, the number of people engaging in the survivorship strategies, and any challenges in the implementation processes.

Qualitative interviews will be conducted with nurses and other healthcare professionals involved in implementation (*n* = 8–10 per community) to understand acceptability, feasibility, barriers, and facilitators to the implementation process. Focus groups will also be conducted with cancer survivors and families from each community that participated in field testing the intervention (total of 2–4 focus groups; 20–40 participants) to understand acceptability of the survivorship strategies, barriers, and supports to participating in them, and perceived ways in which the strategies promote healing, cancer screening, and psychosocial support.

All qualitative data including site visit field notes will be entered into qualitative software (NVIVO 12) to facilitate data management and analysis. Data will be analyzed for themes within specific communities and collectively across all communities and data. The process log will descriptively document the implementation processes.

### Outcomes and data collection

The primary outcome of this study is the feasibility and acceptability of implementing cancer survivorship strategies with Indigenous people. We will evaluate the feasibility of implementation, acceptability of strategies to cancer survivors and caregivers, traditional healers and healthcare providers, and perceived influence on healing, cancer screening, and psychosocial support to cancer survivors and caregivers.

### Analysis

We will involve our Indigenous partners in all stages of the analysis, holding regular group meetings to facilitate accurate interpretation of data and iteratively co-develop themes that reflect culturally relevant community beliefs. Trustworthiness of findings will be sought through prolonged engagement with community partners and collaboratively analyzing data. Researchers will explicitly reveal their predispositions and maintain an audit trail and codebook to ensure transparency and dependability of the analytical process.

Data from all phases of this study will be synthesized in order to develop a toolkit for implementing cancer survivorship strategies and supports across Ontario. We anticipate overall findings to include a series of promising practices to support cancer survivorship for Indigenous people including survivorship strategies, characteristics of collaborative partnerships, adaptation and implementation processes, and barriers and facilitators to implementation. The toolkit will consist of a paper-based manual, videos (developed in previous studies), discussion points, and questions for use in different Indigenous communities across Ontario.

## Discussion

This study is part of a larger program of research that explores the process of integrating Indigenous ways of knowing with western research knowledge to address the disparities in healthcare delivery and outcomes among Indigenous people in Canada. To ensure a comprehensive understanding, our team is studying the collaborative processes to translate Indigenous knowledge into culturally safe and responsive strategies and approaches. Through our partnerships, we are using a strength-based approach with people from urban, rural, and remote Indigenous communities to reorient healthcare delivery.

Results of this study will advance knowledge about implementing culturally safe cancer survivorship supports with Indigenous people to improve healthcare services, quality of life, and ultimately health outcomes. As a way forward, findings will inform a large-scale implementation study to reduce healthcare disadvantages and disparities within Indigenous communities across Canada.

## References

[1] Health Council of Canada (2005). The health status of Canada’s first nations, Inuit and Métis peoples.

[2] Canadian Partnership Against Cancer (2013). First Nations cancer control in Canada baseline report.

[3] Canadian Partnership Against Cancer (2009). Report on National Forum on First Nations, Inuit and Métis cancer control.

[4] Canadian Partnership Against Cancer, First Nations, Inuit and Métis Action Plan on Cancer Control. Toronto, Ontario; 2011.

[5] Chiefs of Ontario, Cancer Care Ontario and Institute for Clinical Evaluative Sciences. Cancer in First Nations People in Ontario: Incidence, Mortality, Survival and Prevalence. Toronto, Ontario; 2017.

[6] Kewayosh A, Marrett L, Aslam U, Steiner R, Moy Lum-Kwong M, Imre J (2015). Improving health equity for First Nations, Inuit and Metis people: Ontario’s Aboriginal cancer strategy II. Healthc Q.

[7] Nishri ED, Sheppard AJ, Withrow DR, Marrett LD (2015). Cancer survival among First Nations people of Ontario, Canada (1968–2007). Int J Cancer.

[8] Marrett LD, Chaudhry M (2003). Cancer incidence and mortality in Ontario First Nations, 1968-1991 (Canada). Cancer Causes Control.

[9] Tjepkema M, Wilkins R (2011). Remaining life expectancy at age 25 and probability of survival to age 75, by socio-economic status and aboriginal ancestry. Health Rep.

[10] Canadian Institutes of Health Research (2015). CIHR Institute of Aboriginal peoples’ health strategic plan 2014–18. wellness, strength and resilience of First Nations, Inuit and Métis peoples: moving beyond health equity.

[11] Statistics Canada (2013). Aboriginal peoples in Canada: First Nations people, Métis and Inuit.

[12] Cancer Care Ontario, Aboriginal Cancer Strategy III 2015–2019. Toronto, Ontario; 2015.

[13] Assembly of First Nations (2015). First Nations action plan on continuing care.

[14] MacMillan HL, MacMillan AB, Offord DR, Dingle JL (1996). Aboriginal health. Can Med Assoc J.

[15] Witham H (2000). Remote and rural nursing: an endangered profession?. Aust Nurs J.

[16] Poudrier J, Mac-Lean RT (2009). ‘We’ve fallen into the cracks’: aboriginal women’s experiences with breast cancer through photovoice. Nurs Inq.

[17] Cavanagh BM, Wakefield CE, McLoone JK, Garvey G, Cohn RJ (2016). Cancer survivorship services for indigenous peoples: where we stand, where to improve? A systematic review. J Cancer Surviv.

[18] Thomas R, Gifford W, Poudrier J, Hamilton R, Brooks C, Scott T (2015). First Nations, Inuit, and Métis women's experiences of cancer survivorship: protocol for the national picture project. Int J Qual Methods.

[19] Thomas R, Hammond C, Gifford W, Poudrier J, Brooks C, Hamilton R (2014). Creating with collaborators: A foundation for an arts-based project with First Nations, Inuit and Métis women with cancer.

[20] Hammond C, Thomas R, Gifford W, Poudrier J, Brook C, Hamilton R (2015). The importance of intersecting social contexts to wholistic care for First Nations, Inuit, and Métis women with cancer.

[21] Brooks C, Poudrier J, Thomas-MacLean R, Liamputtong P (2008). Creating collaborative visions with Aboriginal women: a photovoice project. Doing Cross-Cultural Research.

[22] Thomas-MacLean R, Poudrier J, Brooks C (2008). Envisioning the future with young Aboriginal breast Cancer survivors. Atlantis.

[23] Gifford W, Thomas R, Poudrier J, Hamilton R, Scott T, Warner D (2014). Arts based knowledge translation: Understanding First Nations, Inuit and Métis women’s experiences with cancer.

[24] Hammond C, Thomas R, Gifford W. Extending the metaphor of “Two-eyed Seeing” into health research: Reflections on an arts-based project on Indigenous Cancer Experiences. ALL RESEARCH IS A STORY: RECLAIMING INDIGENOUS RELATIONSHIPS IN ACADEMIA A Student-Centred Conference hosted by Carleton University’s Centre for Indigenous Research, Culture, Language and Education (CIRCLE); Ottawa. 2015.

[25] Hammond C, Thomas R, Gifford W, Poudrier J, Scott T, Brooks C (2015). First Nations, Inuit, and Métis women with cancer: The importance of intersecting social contexts and holistic care.

[26] Grimshaw JM, Eccles MP, Lavis JN, Hill SJ, Squires JE (2012). Knowledge translation of research findings. Implement Sci.

[27] Kothari A, Wathen CN (2017). Integrated knowledge translation: digging deeper, moving forward. J Epidemiol Community Health.

[28] Smylie J, Kaplan-Myrth N, Tait C, Martin CM, Chartrand L, Hogg W (2004). Health sciences research and Aboriginal communities: pathway or pitfall?. J Obstet Gynaecol Can.

[29] Estey E, Smylie J, Macaulay A. Aboriginal knowledge translation: understanding and respecting the distinct needs of Aboriginal communities in research. Ottawa; 2009.

[30] Grimshaw J (2010). A guide to knowledge synthesis: a knowledge synthesis chapter.

[31] Graham ID, Logan J, Harrison MB, Straus SE, Tetroe J, Caswell W (2006). Lost in knowledge translation: time for a map?. J Contin Educ Health Prof.

